# Tobacco smoking trends in Samoa over four decades: can continued globalization rectify that which it has wrought?

**DOI:** 10.1186/s12992-017-0256-2

**Published:** 2017-06-12

**Authors:** Christine Linhart, Take Naseri, Sophia Lin, Richard Taylor, Stephen Morrell, Stephen T McGarvey, Dianna J Magliano, Paul Zimmet

**Affiliations:** 1 School of Public Health and Community Medicine, University of New South Wales, Sydney, Australia; 2 Ministry of Health, Apia, Samoa; 3 International Health Institute, Department of Epidemiology, Brown University School of Public Health, Providence, USA; 4 Baker IDI Heart and Diabetes Institute, Melbourne, Australia

**Keywords:** Smoking, Tobacco, Social Determinants of Health, Education, Population Studies

## Abstract

**Background:**

The island country of Samoa (population 188,000 in 2011) forms part of Polynesia in the South Pacific. Over the past several decades Samoa has experienced exceptional modernization and globalization of many sectors of society, with noncommunicable diseases (NCD) now the leading cause of morbidity and mortality. The evolution of risk factor prevalence underpinning the increase in NCDs, however, has not been well described, including tobacco smoking which is related to cardiovascular disease, lung cancer, and chronic obstructive pulmonary disease.

**Methods:**

The present study examines tobacco smoking in relation to different forms and effects of globalization in Samoa using 7 population-based surveys (*n* = 9223) over 1978–2013.

**Results:**

The prevalence of daily tobacco smoking steadily decreased over 1978–2013 from 76% to 36% in men, and from 27% to 15% in women (*p* < 0.0001 both sexes). During 1991–2013, current tobacco smoking also steadily decreased from 64% to 40% in men (*p* < 0.0001), and from 21% to 17% in women (*p* < 0.05). Declines were similar in younger (25–44 years) and older (45–64 years) men and women. Colonial globalization facilitated the introduction and prolific spread of tobacco trade and consumption in the Pacific Islands from the sixteenth century, with many populations inexorably pulled into trade relations and links to the global economy. It has also been a different globalization which may have led to the decline in smoking prevalence in Samoa in recent decades, through global dissemination since the 1950s of information on the harmful effects of tobacco smoking derived from research studies in the USA and Europe.

**Conclusions:**

Over the past 35 years tobacco smoking has steadily declined among Samoan adults; the only NCD risk factor to demonstrate marked declines during this period. By 2013 tobacco smoking in women had decreased to levels similar to Australia and New Zealand (ANZ), however in men smoking prevalence remained more than three times higher than ANZ. The impact on smoking prevalence of the variety of tobacco control interventions that have been implemented so far in Samoa need to be evaluated in order to determine the most effective initiatives that should be prioritized and strengthened.

## Background

Samoa forms part of Polynesia in the South Pacific region, with a population of 188,000 at the 2011 Census [1]. Since the mid-twentieth Century, Samoa has experienced the demographic and epidemiological transitions, characterized by declines in mortality, particularly infant and under-five deaths, and a change in major causes of death from infection and under-nutrition to non-communicable disease (NCD) [2]. The evolution of risk factor prevalence underpinning the evolution of NCD in Samoa has been partly documented by examination of increasing secular trends over 1978–2013 in obesity and type 2 diabetes mellitus (T2DM) prevalence [3] and T2DM incidence [4]. Another important risk factor for NCD is tobacco smoking, which contributes significantly to cardiovascular disease (CVD) [5], lung cancer and chronic obstructive pulmonary disease [6]. Tobacco smoking is the single greatest cause of preventable mortality globally, responsible for approximately 6 million deaths every year [7].

Tobacco has a long history in the Pacific Islands. It was during European maritime global exploration and expansion in the sixteenth and seventeenth centuries that tobacco, a plant native to North America, was first brought to the Pacific region [8]. It soon became one of the most sought after goods merchants offered to Pacific Islanders, pulling these populations inexorably into trade relations and links with the global economy [8]. In 1955 the Fiji Tobacco Company pioneered cigarette manufacturing in the Pacific Islands, with several factories opening throughout the region in subsequent decades – including Samoa in 1979. From the 1950s–1960s however, information derived from medical and epidemiological research studies on the harmful effects of tobacco smoking was widely disseminated globally; especially following the 1956 publication by Doll and Hill in the British Medical Journal [6], the 1962 report by the Royal Collegue of Physicians [9], and the US Surgeon-Generals Report in 1964 [10]. Within populations this information on the harmful effects of smoking was initially disseminated primarily through educated groups, and incorporated into medical care and health promotion by the medical profession, health departments and non-government organizations. Whilst subsequent declines in tobacco smoking have been well documented in many high-income countries [11, 12], long term trends in lower and middle income countries have not been as well described, particularly in the Pacific Island region. The extent to which the ‘globalization’ of knowledge on the harmful effects of tobacco smoking penetrated the Pacific Island region, as successfully as globalization of tobacco itself from the sixteenth century, has yet to be determined.

Over the past three decades, several surveys measuring the prevalence of tobacco smoking have been conducted in Samoa. Establishing population trends over time is more informative than a prevalence measure from a single cross-sectional survey. Data from multiple surveys also smooths variations from unmeasured and unadjusted biases and confounding; augments participant numbers for analysis (greater statistical power); weights studies by their sample size; and increases generalizability by including multiple studies from different periods and sites. Previous attempts at comparing tobacco smoking data from cross-sectional surveys in Samoa have been hindered by differences in the survey questions used to measure tobacco smoking, the inclusion/exclusion criteria used to select survey participants, and the aggregations (age/sex) used for the presentation of tabulated results.

Through access to original survey unit record data, the present study establishes population trends in current and daily tobacco smoking by sex and age group in Samoans aged 25–64 years from seven cross-sectional surveys conducted over 35-years, 1978–2013. The association of education with tobacco smoking over 1991–2013 is also examined. As the surveys differed in the way tobacco smoking was measured, some asked only about current smoking (daily and non-daily smokers combined) and others only about daily smoking, the present study established trends in current tobacco smoking over 1991–2013, and daily tobacco smoking over 1978–2013. This is the first study to use empirical country data to establish nationally representative population trends in tobacco smoking over three decades in Samoa by age and sex; and determine if education (measured by years in primary and secondary school) have an effect on smoking prevalence. This study covers a period of exceptional transformation and modernization of many sectors of Samoan society, particularly patterns of everyday life.

## Methods

### Survey selection

Surveys measuring tobacco smoking in Samoans aged 25–64 years during 1950–2015 were identified through: (1) a literature search of Medline, PubMed and Global Health; (2) an internet search for reports; (3) direct contact with representatives from the Samoa Ministry of Health, the World Health Organization (WHO), and the Secretariat for the Pacific Community; and (4) direct contact with researchers who had conducted studies in Samoa on tobacco use. Current tobacco smoking was defined as smoking any tobacco product in the previous 12 months (the same definition as used in the WHO STEPS surveys) [13]. Daily tobacco smoking was defined as smoking at least one tobacco product on a daily basis. Surveys were included in this analysis if they were nationally representative at the time of the survey, or could be adjusted to the nearest previous census (by age, sex, urban/rural residency) in order to increase national representativeness, reduce selection bias, and minimize heterogeneity between surveys.

Surveys included in analyses were: the 1978 Non-Communicable Disease Risk Factor (NCDRF) Survey (*n* = 1107) [14] and the 13-year repeat survey in 1991 (*n* = 1590) [15]; the Samoan Adiposity and Cardiovascular Disease Risk Factor (SACRF) longitudinal study conducted in 1991 (*n* = 746) and 1995 (*n* = 720) [16]; the 2002 (*n* = 2613) [17] and 2013 (*n* = 1763) [13] Samoa STEPS surveys; and the Samoan Family Study of Overweight and Diabetes (SFSOD) survey conducted in 2003 (*n* = 684) [18]. A combined unit record data set was assembled by concatenating the seven surveys (*n* = 9223).

Several tobacco smoking prevalence surveys undertaken in Samoa were not included in the current study because they were conducted exclusively in a rural sample [19], or in a specific or narrow age group [20–22]. These surveys could not be adjusted to resemble a nationally representative demographic distribution.

### Data collection and categorization of tobacco smoking

Tobacco smoking data in all surveys was collected by individual interview at the survey site and recorded on pre-structured survey forms. The 1978 and 1991 NCDRF surveys asked participants if they were a: (1) non-smoker; (2) ex-smoker; (3) currently smoking up to 20 cigarettes a day; or (4) currently smoking ≥20 cigarettes a day. The 1991 and 1995 SACRF surveys and the 2003 SFSOD survey asked participants if they were currently smoking cigarettes, but did not ask whether consumption was daily. The 2002 and 2013 WHO STEPS surveys asked participants if they currently smoked any tobacco products such as cigarettes, cigars or rolled tobacco; and participants who answered ‘yes’ were then asked if they smoked daily, and the quantity of daily consumption. Four surveys (1991 and 1995 SACRF surveys; 2002 and 2013 STEPS surveys) enabled assessment of smoking prevalence and education based on self-reported years of education (excluding years of preschool education).

### Demographic adjustment and trend analyses

In order to improve national representativeness and minimize selection bias, each survey was variously adjusted to the most recent previous census for age group and urban-rural distributions by sex, using case weights derived from the ratio of the population proportions from the census and the survey for each stratum. This is similar to the methodology used in the WHO STEPS surveys [13]. The prevalence of current and daily tobacco smoking in each survey was then calculated by sex and age group; and the prevalence of smoking ≥20 tobacco products/day was calculated by sex only due to small numbers. Trends in tobacco smoking prevalence over the period were examined using binomial regression, and a logarithmic function was fitted to display the period trend line as this was determined to best describe the data.

Mean years of education in non-smokers compared with current and daily smokers was calculated, and statistical significance of differences between the sex-specific strata within each survey was assessed using the Student’s t-test. Binomial regression was used to investigate the effect of increasing years of education on the likelihood of current tobacco smoking for 1991–2013 by comparing the period trend in the relative risk (RR) of current tobacco smoking adjusting for age, with that of the period trend adjusting for age and years of education (as continuous variables). The period effect assessed in this study is a measure of the outcome of all influences which promote or lead to decline in tobacco smoking in the population over time. SAS version 9.4 was used for analysis (SAS Institute Inc., Cary, NC, USA).

## Results

### Current tobacco smoking

**Fig. 1:**
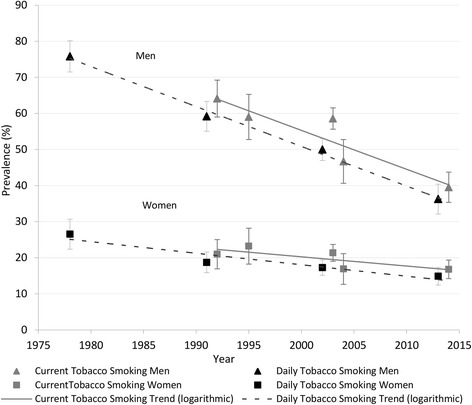
Prevalence of current and daily tobacco smoking (%), by sex, Samoa, 1978–2013^. FOOTNOTES: ^ Prevalence data adjusted to the most recent previous census for age group and urban-rural distributions by sex to improve representativeness; Current Smoker (daily and non-daily smokers combined);Daily Smoker (consumes ≥1 tobacco product/day); where multiple data points overlap markers have been offset on the graph to improve interpretation; vertical straight lines through point estimates represent 95% statistical confidence intervals. A logarithmic function was fitted to display the trend line as this was determined to most appropriately describe the data

Over 1991–2013, current tobacco smoking declined steadily from 64.1% to 39.5% in men (*p* < 0.0001); and from 21.0% to 16.8% in women (*p* < 0.05) (Table 1 and Fig. 1). Declines were greater in older (45–64 years) compared with younger (25–64 years) age groups in both sexes (Table 2).

**Table 1:** Prevalence of current and daily tobacco smoking (%), by sex, Samoa, 1978–2013^a^

Year	N	Current Smoker	Daily Smoker	≥20/day	N	Current Smoker	Daily Smoker	≥20/day
	Men				Women			
1978	492	-	75.8 (71.5–80.2)	15.6 (12.0–19.2)	615	-	26.5 (22.4–30.7)	1.12 (0.17–2.07)
1991a	703	-	59.2 (55.0–63.4)	14.3 (11.6–17.1)	887	-	18.7 (15.8–21.6)	3.00 (1.71–4.27)
1991b	355	64.1 (59.0–69.2)	-	-	391	21.0 (16.9–25.0)	-	-
1995	343	50.0 (52.7–65.3)	-	-	377	23.2 (18.3–28.2)	-	-
2002	1202	58.6 (55.6–61.6)	50.0 (47.0–53.0)	16.2 (13.9–18.4)	1411	21.3 (19.0–23.7)	17.3 (15.1–19.4)	3.09 (2.15–4.01)
2003	317	46.7 (40.6–52.7)	-	-	367	16.9 (12.6–21.2)	-	-
2013	700	39.5 (35.3–43.7)	36.3 (32.1–40.4)	6.98 (4.88–9.08)	1063	16.8 (14.2–19.4)	14.9 (12.4–17.3)	3.16 (1.95–4.36)
*5-yr. change ∞*		−5.89*** (−7.28|-4.51)	−5.47*** (−6.20|-4.74)	−0.88* (−1.47|-0.28)		−1.26* (−2.27|-0.26)	−1.46*** (−2.00|-0.93)	0.30* (0.07|0.53)

*p < 0.05 **p < 0.001 ***p < 0.0001; N (number of participants in stratum); 95% confidence intervals in brackets below point estimates; Current Smoker (daily and non-daily smokers combined); Daily Smoker (consumes ≥1 tobacco product/day); ≥20/day (consumes ≥20 tobacco products/day); 5-yr. change∞ (represents the change in prevalences from binomial regression in each 5 year period). ^a^Prevalence data variously adjusted to the most recent previous census for age group and urban-rural distributions by sex using case weights derived from the ratio of the population proportions from the census and the survey for each stratum to improve representativeness. Surveys included in analyses are: 1978 NCDRF Survey [14]; 1991a NCDRF Survey [15]; 1991b SACRF longitudinal Study [16]; 1995 SCARF Study [16]; 2002 WHO STEPS Survey [17]; 2003 SFSOD Survey [18]; 2013 WHO STEPS Survey [13]. Unit record survey data included in the present study were provided by original survey researchers in 2015

**Table 2:** Prevalence of tobacco smoking (%), by age-group, Samoa, 1978–2013^a^

Year	N	25–44 years	N	45–64 years	N	25–44 years	N	45–64 years
	Men				Women			
Current Tobacco Smoking								
1991b	255	60.6 (54.4–66.7)	100	71.6 (62.4–80.8)	276	20.8 (16.0–25.6)	112	21.4 (13.6–29.1)
1995	208	56.9 (48.7–65.1)	135	63.4 (54.0–72.8)	224	22.7 (16.3–29.1)	153	24.3 (16.6–32.1)
2002	745	58.3 (54.5–62.0)	457	59.2 (54.4–64.1)	860	21.7 (18.8–24.6)	551	20.7 (16.9–24.5)
2003	186	48.5 (41.0–56.1)	131	45.1 (35.9–54.3)	216	15.8 (10.5–21.1)	151	19.0 (12.2–25.9)
2013	357	42.5 (36.7–48.3)	343	34.1 (28.5–39.7)	592	17.5 (14.1–20.9)	471	15.5 (11.8–19.1)
*5-yr. change ∞*		−4.16*** (−6.00|-2.31)		−8.91*** (−11.0|-6.81)		*−1.08 (−2.37|0.22)*		−1.98* (−3.57|-0.40)
Daily Tobacco Smoking								
1978	252	76.9 (71.2–82.7)	240	75.1 (69.1–81.1)	335	21.8 (16.6–27.0)	280	35.2 (28.5–41.9)
1991a	376	59.5 (54.2–64.8)	327	59.6 (53.7–65.5)	489	18.3 (14.7–21.9)	398	20.4 (15.6–25.3)
2002	745	49.7 (45.9–53.5)	457	50.1 (45.2–55.0)	860	17.9 (15.2–20.6)	551	16.1 (12.6–19.6)
2013	357	38.4 (32.7–44.1)	343	32.3 (26.8–37.9)	592	15.3 (12.0–18.5)	471	14.1 (10.6–17.6)
*5-yr. change ∞*		−5.42*** (−6.44|-4.40)		−5.87*** (−6.91|-4.82)		−0.84* (−1.56|-0.11)		−2.60*** (−3.39|-1.82)

*p < 0.05 **p < 0.001 ***p < 0.0001; N (number of participants in stratum); 95% confidence intervals in brackets below point estimates; Current Smoker (daily and non-daily smokers combined); Daily Smoker (consumes ≥1 tobacco product/day); 5-yr. change∞ (represents the change in prevalences from binomial regression in each 5 year period). ^a^Prevalence data variously adjusted to the most recent previous census for urban-rural distributions by sex using case weights derived from the ratio of the population proportions from the census and the survey for each stratum to improve representativeness. Surveys included in analyses are: 1978 NCDRF Survey [14]; 1991a NCDRF Survey [15]; 1991b SACRF longitudinal Study [16]; 1995 SCARF Study [16]; 2002 WHO STEPS Survey [17]; 2003 SFSOD Survey [18]; 2013 WHO STEPS Survey [13]. Unit record survey data included in the present study were provided by original survey researchers in 2015

### Daily tobacco smoking and smoking ≥20 tobacco products/day

Over 1978–2013, daily tobacco smoking steadily declined from 75.8% to 36.3% in men (*p* < 0.0001); and from 26.5% to 14.9% in women (*p* < 0.0001) (Table 1 and Fig. 1). Similar to current tobacco smoking, the declines were greater in older compared with younger age groups in both sexes. Smoking ≥20 tobacco products/day declined in men from 15.6 to 7.0% (*p* < 0.05), whilst in women there was a small but significant absolute increase from 1.1% to 3.1% (*p* < 0.05) (Table 1).

### Mean years of education in non-smokers compared with current and daily smokers

Throughout 1991–2013, among men, mean years of education was higher in non-smokers compared to current smokers. The difference between the groups did not exceed one year, but was statistically significant (*p* < 0.05) in all surveys except the 1995 SACRF survey. In women, the reverse was found with mean years of education at parity or higher in current smokers compared to non-smokers throughout 1991–2013. As with men, the difference between the groups was small and did not exceed one year, with the difference only reaching statistical significance (*p* = 0.01) in 2013 with 11.7 mean years of education among female non-smokers and 12.3 among current smokers (Table 3).

**Table 3:** Effect of mean years of education on tobacco smoking prevalence, by sex, Samoa, 1991-2013^a^

	Non-smoker		Current smoker			Daily smoker		
MEN	N	Years Education	N	Years Education	*p*-value	N	Years Education	*p*-value
1991b	125	9.8 (9.3–10.2)	221	9.1 (8.7–9.6)	**0.038**	-	-	-
1995	109	9.5 (9.0–10.0)	153	9.0 (8.6–9.4)	0.159	-	-	-
2002	512	10.9 (10.6–11.2)	674	10.3 (10.0–10.5)	**<0.001**	576	10.2 (9.9–10.5)	**<0.001**
2013	342	11.9 (11.5–12.2)	215	11.1 (10.7–11.5)	**0.006**	198	11.1 (10.7–11.5)	**0.007**
WOMEN								
1991b	301	10.0 (9.7–10.2)	81	10.3 (9.8–10.8)	0.216	-	-	-
1995	220	10.0 (9.7–10.3)	66	10.6 (10.0–11.2)	0.087	-	-	-
2002	1087	11.0 (10.8–11.2)	308	11.0 (10.7–11.2)	0.535	249	11.0 (10.7–11.3)	0.773
2013	706	11.7 (11.5–11.9)	143	12.3 (11.8–12.7)	**0.012**	127	12.3 (11.9–12.8)	**0.009**
Relative risk (RR) of current versus non-smoker, 1991–2013^b^								
			MEN		WOMEN			
1. Period			0.78 (0.73–0.83)		0.94 (0.90–0.98)			
2. Period and age			0.78 (0.73–0.83)		0.94 (0.90–0.98)			
Change in period RR (1–2)^c^			0.10%		0.05%			
3. Period and age and education			0.81 (0.76–0.87)		0.93 (0.89–0.97)			
Change in period RR (2–3)^c^			−4.55%		+1.32%			

N (number of participants in stratum); Years Education (mean years of education in stratum); 95%CI confidence intervals in brackets next to estimate of mean years of education in each stratum and odds ratio; ^a﻿^prevalence data adjusted to the most recent previous census for age group and urban-rural distributions by sex to improve representativeness﻿﻿. ^b^relative risk (RR) of being a current versus non-smoker in 1991 compared to 2013; where the RR for current smoker in 1991 is 1.0, and the RR displayed in the table is the RR in 2013. ^c^proportional change from the previous model. Current Smoker (daily and non-daily smokers combined); Daily Smoker (consumes ≥1 tobacco product/day); *p*-values in bold represent statistical significance at *p* < 0.05. Surveys included in analyses are: 1991b SACRF longitudinal Study [16]; 1995 SCARF Study [16]; 2002 WHO STEPS Survey [17]; 2013 WHO STEPS Survey [13]. Unit record survey data included in the present study were provided by original survey researchers in 2015

In men, higher education reduces the period effect of current tobacco smoking decline over 1991–2013 from 22% to 19% (RR of 0.78 adjusting for age, compared to 0.81 adjusting for age and education). In women, however, higher education contributes to the higher prevalence of smoking, to the extent that the period effect would have been a 7% decline rather than the observed 6% (RR 0.94 adjusting for age, compared to 0.93 adjusting for age and education) (Table 3). The period decline in smoking prevalence remained statistically significant (*p* < 0.05) in both sexes after adjusting for age, and after adjusting for age and education. Education was significant in the regression model in men (*p* < 0.001) and women (*p* < 0.05); whilst age was not statistically significant in either sex.

## Discussion

Empirical survey data for Samoans aged 25–64 years demonstrates a steady decline in both sexes in the prevalence of daily tobacco smoking over 1978–2013, and in current tobacco smoking over 1991–2013. The prevalence of daily tobacco smoking in 2013 among Samoan women (15% in the 25–64 age group) was similar to levels in Australia (12% in ≥18 years in 2013) [23] and New Zealand (14% in ≥15 years in 2014/15) [24]. Among Samoan men however, the prevalence was substantially higher (40% in the 25–64 age group) compared to Australia (15% in ≥18 years in 2013) [23] and New Zealand (16% in ≥15 years in 2014/15) [24]. In men, higher levels of education were associated with lower levels of smoking prevalence throughout the period; whilst in women, there was no significant difference until 2013, at which point current smokers had significantly more years of education than non-smokers.

Several tobacco smoking prevalence surveys undertaken in Samoa were not included in the present study because they could not be adjusted to resemble a nationally representative demographic distribution. The Samoa Demographic and Health Surveys (DHS) undertaken in 2009 [21] and 2014 [22] found that current tobacco smoking in participants aged 25–44 years ranged from 39 to 49% in 2009 and 45–47% in 2014 in men, and from 16 to 20% in 2009 and 14–18% in 2014 in women; similar to findings in the present study of 43% in men and 18% in women in 2013 in the same age group (25–44 years). An estimation of tobacco and cigarette consumption can also be obtained from household expenditure surveys; however, using proportions of household expenditure on tobacco as a surrogate for tobacco consumption can be misleading. For example, a large increase in the tobacco excise, or a rise in price due to tobacco crop failures, can produce an apparent increase in the tobacco proportion of household expenditure without an increase, or even a decrease, in tobacco actually consumed. Bearing in mind these limitations, estimates of household expenditure on tobacco as a percentage of total household expenditure (including cash expenditure, subsistence and gifts) has shown a decline in Samoa, from 1.5% of total household expenditure in 2002 [25], to 1.4% in 2008 [26], and 0.3% by 2013–14 [25]. Whilst not directly comparable, the findings of these studies are congruent with those in the present study.

Tobacco has a long history in the Pacific Islands, dating back to the seventeenth century in Papua New Guinea and Guam, however, its arrival in the rest of the region was much later [8]. Samoa is thought to have had little contact with Westerners until the arrival of the missionary John Williams in 1830 [27], and tobacco may not have been introduced to Samoa until after this time [8]. There are few reliable and detailed data to describe the interisland trade of tobacco among Samoa and neighboring countries such as Fiji and Tonga during the nineteenth century. However, there is evidence that tobacco was readily and rapidly incorporated as an important exchange commodity in these traditional trade networks [8]. In 1955 Fiji became the first country in the region to manufacture cigarettes [28], and in 1979 Samoa also began commercial manufacturing.

When tobacco smoking in Samoa reached its peak prevalence has not been determined, however the present study shows that since the first national survey in 1978 the trend has steadily declined in both sexes, albeit from an extremely high baseline. There was very little active opposition to tobacco smoking in the Pacific Islands before the 1960s, and efforts in Samoa prior to this time were primarily based on moral standings of the Christian church espousing abstinence from tobacco, alcohol and narcotics [8]. During the 1950s the first robust medical evidence of the harmful effects of tobacco smoking was produced from prospective cohort studies in the UK [6] and the USA [29, 30]. In the early 1960s a report from the Royal College of Physicians of London [9] stated that cigarette smoking was associated with coronary heart disease and lung cancer, and two years later the first US Surgeon General’s Report on Smoking and Health stated that the association was causative [10]. This information was widely disseminated globally, and smoking prevalence subsequently declined in many high-income countries, including Australia and New Zealand [11, 12]. The extent to which this information from the UK and the USA was disseminated throughout the Samoan population during the 1960s has not been documented in detail, however it was reported in the Pacific Island Monthly news bulletin in 1964 that cigarettes had suffered adverse publicity in Samoa after the US Surgeon General’s report was given full publicity the month after it was released in the US [31].

The initial principal recipients in Samoa of the evidence of negative health effects from tobacco smoking were most likely the medical profession. The connections between Anglophone physicians, particularly from the UK, with the Pacific Island region during that period is exemplified by the lead researcher in the British Doctors Study [6], Sir Richard Doll, co-authoring on the first publication of cancer incidence in Fiji (over 1965–69) from the newly established cancer registry; which compared cancer incidence in Fiji (including lung cancer) with that of Birmingham, England [32]. Resistance to tobacco smoking by the medical profession in Samoa was documented in 1978 when the Chief Surgeon at Apia’s National hospital expressed concerns about the health impacts of smoking at public protests against the establishment of a cigarette factory in Samoa [33]. However, the Government did not act upon this knowledge, and despite public protests and advocates from the medical profession expressing opposition, the factory was permitted to proceed the following year. The push from British and American tobacco corporations to expand cigarette manufacturing in the region, including Samoa, on the grounds of significant economic benefit to Pacific Island countries, likely hampered these early efforts at tobacco control [8].

The hesitation of Pacific Island Governments, policy-makers and legislators to strongly discourage or oppose tobacco smoking during the 1960s–1970s was likely influenced by perceived adverse economic impacts. By the early 1970s the tobacco industry in Fiji had grown to constitute 6% of the national economy and was one of the nation’s largest manufacturing units, staffed almost exclusively by local workers [34]. Whilst in Samoa, the Government remained a 40% shareholder in the country’s tobacco factory from its establishment in 1978 until 2000 [35]. In the absence of any strong legislative opposition or anti-smoking policy however, a decline in the community sentiment towards tobacco smoking appears to have begun during this period, probably supported by continued medical opposition. During the mid-1960s newspaper articles described expansion of the tobacco industry in the region in terms of economic benefits to the local population [36], whilst during the 1970s articles began to challenge the continued expansion in the region in light of known adverse health effects, with headlines such as ‘Smoking chums have an eye on all the Pacific’ [34], ‘Smoking no wealth hazard in Tonga’ [37], and ‘Apia: smoke of battle’ [33]. From the late 1970s, public-led campaigns against tobacco smoking increased in the region with demands that Governments implement anti-tobacco smoking policy and legislation, including a ban on all cigarette advertising and health warning labels on cigarette packets [38, 39], as was beginning to occur in developed Pacific rim countries. It was not until 1998 however that the first Tobacco Control Bill in the Pacific Island region was endorsed by the Fiji parliament, [40], and in 2003 Fiji became the first developing country to ratify the WHO Framework Convention on Tobacco Control (FCTC).

Samoa ratified the WHO FCTC in 2005, and in 2008 the first Tobacco Control Act was enacted by the Samoan Parliament [41], followed by the Samoa Tobacco Control Regulations in 2013 [42]. Under the Act and Regulations, advertisement and sale of tobacco products is restricted; Government offices, public transport, health facilities, schools and restaurants have designated smoke-free areas; and all cigarette or loose tobacco packaging must have a text health warning covering 30% of the front and back of cigarette packaging [42]. Anti-smoking television advertising and hundreds of billboards with a variety of anti-smoking messages have been used in Samoa in recent years [42]. Culturally relevant, evidence-based initiatives are currently being trialled; a mobile text message based smoking cessation support programme; and the WHO PEN (Package of Essential NCD interventions) *Fa’a Samoa*, which is a village-based NCD screening initiative designed to be implemented by local communities, including women’s groups [43]. The effectiveness of these more recent interventions specifically remains to be seen, as declines in tobacco smoking in Samoan adults began prior to their implementation. More detailed research is needed to quantify the extent that more recent tobacco smoking trends are related to tobacco control measures.

In the absence of accurate cause-specific mortality data from complete vital registration, the modelled estimates reported by the Institute for Health Metrics and Evaluation for 2015 indicate that CVD is the leading cause of death in Samoa, with the period trend in proportional mortality from CVD increasing [44]. This is consistent with analyses of CVD risk factors in Samoa over the past 35 years including hypertension, T2DM and obesity, which have shown a consistent increase [3, 14, 17]. The trends in daily tobacco smoking reported in the present study are the first to indicate a consistent and considerable decrease in a major CVD risk factor in the Samoan population during the past three decades. Population-based case-control data collected in Australia and New Zealand during 1986–1994 as part of the WHO MONICA Project indicated that episodes of a major CVD event (fatal and non-fatal) rapidly reduced within 1–3 years of smoking cessation in both sexes, and returned to a level comparable to a never smoker within 4–6 years [45]. Similar findings were observed from population-based cohort data collected from women during 1980–2004 as part of the Nurse’s Health Study in the US, which indicated a rapid reduction in CVD mortality in the first 5 years after smoking cessation [46]. Decline in mortality from lung cancer and chronic obstructive pulmonary disease (COPD) following smoking cessation is slower. In the Nurse’s Health Study a 21% lung cancer mortality reduction was observed within the first 5 years after cessation, but the mortality risk returned to parity with never smokers only after 25 years; for COPD an 18% mortality reduction was observed 5–10 years after cessation, with the mortality risk reaching parity with a never smoker after 20 years [46].

A potential limitation of the present study is that estimates of tobacco smoking prevalence are based on self-report survey data. In the presence of anti-smoking campaigns and increased public awareness of negative health outcomes from active and passive smoking, the perception of smoking as a socially undesirable behaviour could decrease a participants willingness to self-report such behaviour [47]. A recent systematic review of 67 studies published between 1983 and 2006 examining the relationship between self-reported smoking, and smoking confirmed by cotinine measurement, found that overall smoking prevalence based on self-report was under-estimated by −4.8% to −9.4%, depending on the bodily fluid used to measure cotinine [48]. However, no trend of increasing or decreasing bias was found over the three decades analysed in the review (1983–2006) [48], which is a similar period to the present study (1978–2013). An assessment of the level of potential under-estimation in smoking prevalence based on self-reported data in the Samoa population, or a similar Pacific Island population, has not been undertaken.

## Conclusions

This study is the first to identify trends in the prevalence of current and daily tobacco smoking, and smoking of ≥20 tobacco products a day, in Samoa, by sex and age group using seven large cross-sectional surveys with standard definitions and methodology for analysis. The results indicate that significant reductions have been achieved in current and daily tobacco smoking in the Samoan adult population in recent decades, although prevalences remain high in men in 2013 at almost three-fold that of Australia or New Zealand. Globalization facilitated the introduction and prolific spread of tobacco trade and consumption in the Pacific Island region from the sixteenth century, under various guises of mercantilism and colonialism. It is also globalization, however, which may have pioneered and facilitated the decline in smoking prevalence observed in Samoa in recent decades, through the global dissemination of information since the 1950s on the harmful effects of tobacco smoking derived from research studies in the UK and the USA. And more recently through the WHO Framework Convention on Tobacco Control, a global treaty to which 168 states have become signatories [49].

It is important however that global approaches to address tobacco control in the Pacific region take into account the heterogeneity of these populations and acknowledge the history of tobacco use, and the local cultural meaning and values that surround tobacco, in the populations in which policies and interventions are to be implemented. Further research is needed to evaluate the impact of the variety of tobacco control interventions that have been implemented in the Samoan context over recent decades, in order to determine the most effective interventions that should be prioritized and strengthened to not only sustain consistent declines achieved thus far, but to also reduce the high smoking prevalence in Samoan men.

## Abbreviations

ANZ: Australia and New Zealand
COPD: Chronic obstructive pulmonary disease
CVD: Cardiovascular disease
DHS: Demographic and Health Survey
FCTC: Framework Convention on Tobacco Control
NCD: Non-communicable disease
NCDRF: Non-communicable disease risk factor
RR: Relative risk
SACRF: Samoan adiposity and cardiovascular disease risk factor
SFSOD: Samoan family study of overweight and diabetes
T2DM: Type 2 diabetes mellitus
UK: United Kingdom
USA: United States of America
WHO: World Health Organisation
